# Ten simple rules for typographically appealing scientific texts

**DOI:** 10.1371/journal.pcbi.1008458

**Published:** 2020-12-31

**Authors:** Lars Ole Schwen

**Affiliations:** Fraunhofer Institute for Digital Medicine MEVIS, Bremen, Germany; Dassault Systemes BIOVIA, UNITED STATES

## Introduction

Text is ubiquitous in everyday scientific work—when was the last time you spent 5 minutes working without writing, reading, or interacting with any kind of equipment that had text (scales, labels, brand name, etc.) on it? Most forms of communicating ideas and findings in science are based on text, e.g., BSc/MSc/PhD theses, manuscript drafts, grant proposals, reports, or job applications. In addition, text appears in figures, (electronic) slides for presentations, and posters, i.e., in formats focused more strongly on a graphical presentation.

**Fig 1:**
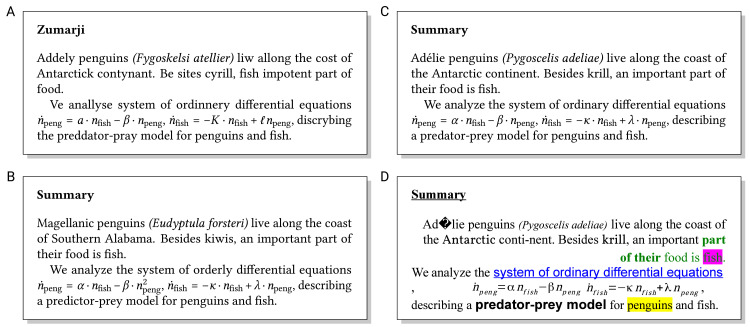
What jumps out to you first? Poor formatting, incorrect language, or wrong contents?

All these documents are usually written to convince the audience of the quality of your ideas or results, ultimately with the goal of a positive evaluation (grading, decision on funding/hiring, etc.). A good visual appearance of the text and graphical elements is key for making a good first impression on the audience. When sustaining this impression by clearly structured and well-written text, professional layout is again important because less-than-optimally typeset texts distract the audience from fully appreciating the high-quality content [1]. Even though single visual inconsistencies cost the readers only a fraction of a second, these interruptions to the flow of reading add up and subconsciously frustrate the readers, possibly undermining your credibility. Poor visual appearance and language can be spotted at first glance in Fig 1, and incorrect content (or a confusing structure, not shown in Fig 1) take much longer to notice. Properly formatting text is particularly challenging in interdisciplinary fields like Computational Biology, where authors are faced with a variety of text elements, e.g., Greek characters, mathematical formulas, chemical formulas, and source code listings. Similar to inconsistent writing style, inconsistent formatting may indicate plagiarism, e.g., stray dashes resulting from copying and pasting hyphenated text, garbled characters, and fonts/formatting copied from the source.

Scientists frequently need to produce final document layout themselves, either from scratch or based on a template—where some templates are well designed and others are, well, “designed.” If a template is given, fewer decisions need to be made, but some typographical knowledge is still helpful to understand the template and to deal with issues unforeseen therein. Ideally, the actual typesetting is subsequently done by trained professionals, e.g., working with publishers, who know what they are doing [2]. Submissions should, in this case, follow the publishers’ guidelines and templates, but still be prepared carefully, as “reviewers’ opinion about a manuscript can be skewed by careless formatting” [3]. Typography is thus one of the tools of the trade for scientists.

This article is meant as a practical guide for typesetting scientific texts, including motivation for the recommendations. While focusing on the intended layout, the rules also provide hints on how these results can be obtained in common text processing/typesetting tools (such as Microsoft Word/LibreOffice Writer, Google Docs, and LaTeX). These rules are meant to complement

- detailed typography textbooks or reference books [4–7] by providing hands-on recommendations for everyday scientific writing;
- software manuals (typically focused on features and how to achieve specific formatting) by explaining which formatting makes sense in which case;
- style manuals [8–10];
- tips for scientific writing [11–17] and collaboration tools [18–21]; and
- specialized recommendations for slides [22,23] and posters [24,25].

The rules primarily apply to English (specifically American English), and many of them also apply to other languages using the Latin alphabet and beyond. However, ligatures and diacritics (Rule 2), punctuation and its spacing (Rule 2), hyphenation (Rule 3), and number formatting (Rule 8) vary between languages.

## Rule 1: Fonts—Choose a suitable (type)face for your work

**Fig 2:**
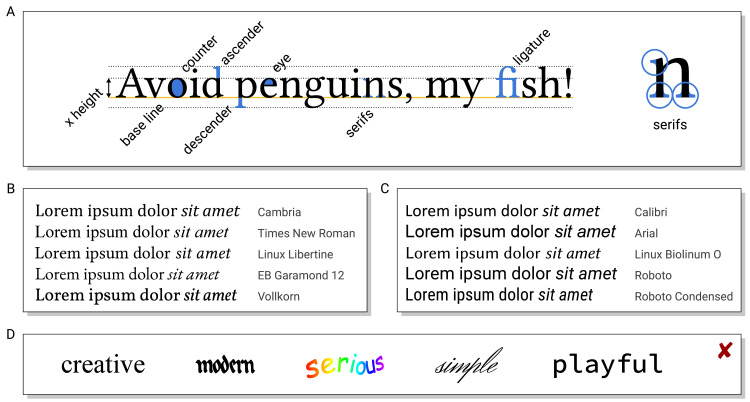
(A) Terminology to describe the “anatomy” of glyphs. (B, C) Samples of serif (B) and sans serif fonts (C), all of them nominally of the same size (but notice the differences in width, x versus ascender/descender height and overall apparent size). (D) Confusing use of fonts for a purpose they were not designed for.

Fonts should be chosen according to the intended function. Documents primarily consisting of text are usually typeset in serif fonts where letters end in horizontal lines (see Fig 2A) guiding the readers’ eyes through the lines like a “railroad track” [26]. Moreover, serifs provide distinctive shapes of words (Fig 2B). This allows more easily reading text by fixing a few points in each line (saccades, [27]) rather than continuously reading each individual letter. These properties generally make serif fonts easily readable.

In contrast, posters, slides, and figure annotations containing only little text and incomplete sentences require each word to be clearly legible. In this case, sans serif fonts are more suitable (Fig 2C). Nonproportional (typewriter-like) fonts where each glyph has the same width have a technical appearance and are used, e.g., for source code listings. Calligraphic, handwritten, or otherwise creative fonts may lack a serious appearance and should be used with care in scientific content, e.g., if a handwritten/sketched look is intended [28]. Besides the function, fonts can convey characteristics like elegant, modern, or traditional (see Fig 2D) [29].

In 1 document, only as many fonts as necessary should be mixed. Fonts should be combined to complement each other with the intended level of contrast and with matching x height and length of ascenders/descenders. The main font for the text should include all required diacritics (e.g., for proper names), non-Latin characters (e.g., Greek), and symbols (e.g., arrows or for mathematical formulas), cf. Rule 2.

## Rule 2: Individual characters and words—Get the details right

Text is composed of single characters including (uppercase and lowercase) letters, numbers, punctuation, characters with diacritics, and symbols. Typographically, however, text is composed of glyphs, representations of characters in a specific shape and design.

Certain combinations of letters appear differently when combined, forming so-called ligatures (e.g., the “fi” in the word fish in Fig 2A). Ligatures enhance readability by avoiding visual gaps inside words and are examples of 1 glyph representing multiple characters.

**Fig 3:**
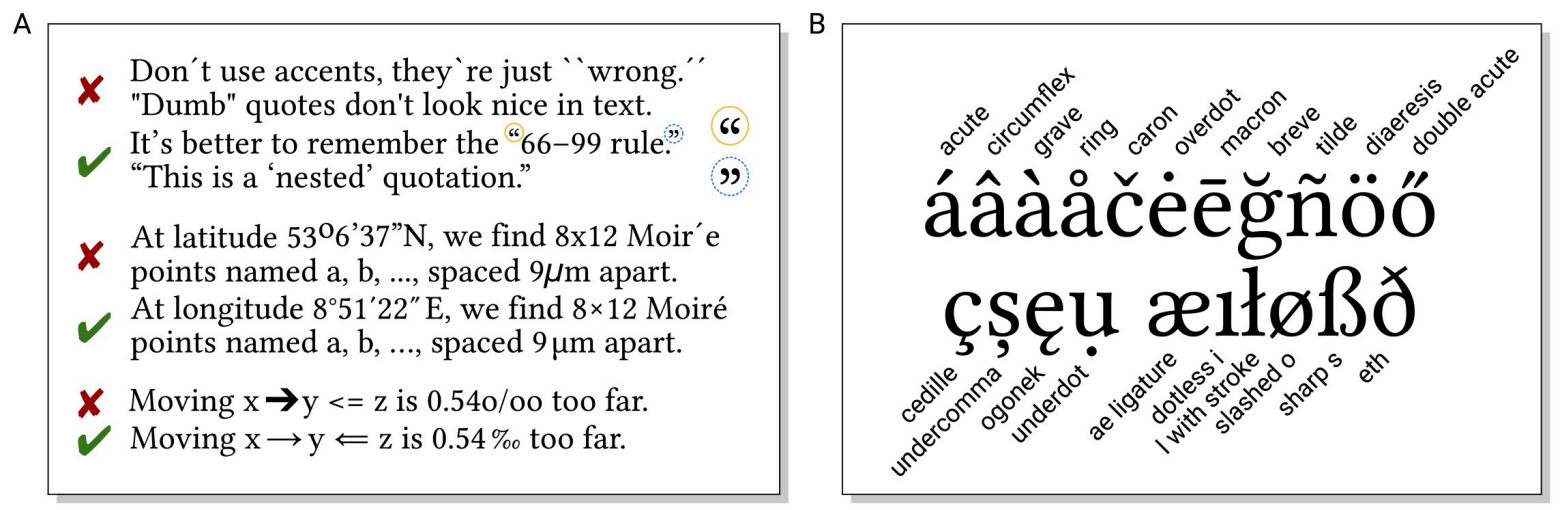
(A) Typographically correct symbols make a difference between sloppily written and conveniently readable texts. (B) Many language use the Latin alphabet combined with different types of diacritics and additional characters.

Punctuation is used to structure sentences and should use correct glyphs (cf. Fig 3A).

Quotation marks exist in 2 forms: straight/dumb (as typed on the keyboard) and typographic form. In English, raised 6/66 and 9/99 forms as shown in Fig 3A are used as opening and closing quotation marks, respectively, depending on whether you follow a style using single or double quotes. Punctuation is placed before or after closing quotation marks depending on whether it is part of the quote, except for periods and commas always placed before the closing quotation mark [10]. Apostrophes have the raised-9 form of a closing single quotation mark. Prime and double prime symbols are used, e.g., for feet/inches, arcminutes/arcseconds in geographic latitude and longitude (cf. Fig 3B), and derivatives in mathematics and to indicate positions of carbon on ribose rings in molecular biology. Neither of these symbols should be confused with accents (see below).

Dashes come in 3 flavors. For hyphenation (see also Rule 3) and compound words, a standard dash (-) is used. The slightly longer en dash (–) is used for ranges (e.g., pages 24–33), sometimes as the symbol in bullet lists (see Rule 6), and to indicate naming after separate persons (e.g., the Michaelis–Menten reaction) as opposed to hyphenated names (e.g., 2008 Nobel laureate Françoise Barré-Sinoussi). The minus sign is typically similar to the en dash. The em dash—as shown here—is used as a phrase marker—or for adding afterthoughts. However, besides unspaced em dashes, spaced en dashes are also recommended for these purposes [7].

Accents and other diacritics (cf. Fig 3B) may be complicated, in particular outside one’s native language. Still, they are worth getting right—imagine what a picky reviewer will think about your scientific work if you cited them, but did not even manage to spell their name correctly.

Correct symbols that cannot directly be typed can be selected/copied from a character table or entered via their respective Unicode code points. Both these options are tedious. Using defined macros or auto-correction features of the text processing software can be more convenient, but do not always work as intended and should be checked.

## Rule 3: Lines and paragraphs—Keep the text flowing

**Fig 4:**
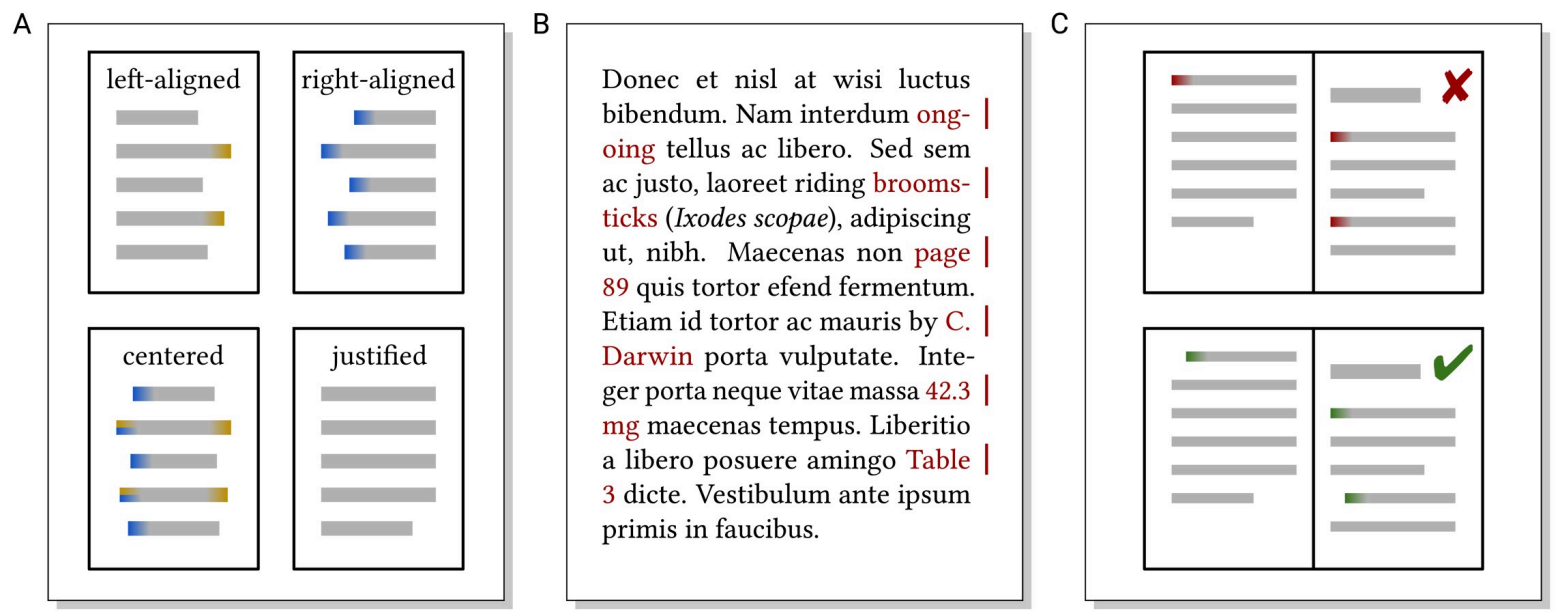
(A) Left-aligned text randomly emphasizes words appearing at the end of the line (indicated in orange); right-aligned text makes finding the next line unnecessarily difficult for the readers (indicated in blue); centered text combines both disadvantages; and justified text avoids these issues and has the calmest appearance. (B) Examples where hyphenation or line breaks interrupt the flow of reading (hyphenation examples from p. 115 in [30]). (C) Indenting the first line of paragraphs (except after headings) clearly indicates where a new paragraph starts, and this may be unclear at the top of a page otherwise.

Paragraphs consist of lines of text (see Rule 5 for a discussion of line width). Paragraphs can be typeset left-aligned, centered, right-aligned, or fully justified; cf. Fig 4A. Justifying text requires aligning both the left and right ends of lines, and this is commonly achieved by stretching the spacing between words. Paragraphs in continuous text are usually typeset justified. This is most convenient to read as paragraph breaks can be spotted easily, and there is no random graphical emphasis on words at the beginning or end of lines which are longer than the surrounding lines. Shorter pieces of text can also be typeset left-aligned, e.g., on posters and slides. Centered and right-aligned text is sometimes used for headings, displayed equations, or tables (cf. Rule 7). Such alignment is not suitable for longer texts as it makes finding the next line inconvenient (Fig 4A).

Line breaks in paragraphs should not interrupt the flow of reading. To prevent undesired line breaks, e.g., between numbers and their unit (Fig 4B), non-breaking spaces should be used.

Words may need to be hyphenated to avoid large gaps in lines in justified text. Text should be hyphenated by the respective feature of the text processing/typesetting software. Automatic hyphenation usually works well if language settings are correct, but should be checked for misleading hyphenations (Fig 4B). Enforcing hyphenation by manually entering dashes and spaces/line breaks may lead to stray dashes when fur-ther editing the text.

The first line of paragraphs is frequently indented (Fig 4C) to clearly indicate that a new paragraph has started (except immediately after headings where indentation would be redundant). In contrast to vertically spacing paragraphs, indenting is also visible after a page break, below a figure, and after lists.

Alignment, indentation, and other formatting of paragraphs should not be applied manually for each paragraph, but via suitably defined paragraph or document styles. Ideally, this is provided by the document template.

## Rule 4: Emphasize what is important, and only that

Not all words in a text are equally important, and some need to be distinguished visually. Visual emphasis, however, should not happen by accident (e.g., because a word happens to appear at the end of a line or because a symbol needs to be used from a different font). Instead, emphasis should result from a conscious decision, and a suitable and consistent way of formatting different types of importance should be used. The main purposes of increased visibility of words are.

- structuring (providing “entry points” on the page where one could start reading),
- emphasis (where stressing something only makes sense within the context), and
- markup (e.g., in bibliographies or for syntax highlighting in source code).

**Fig 5:**
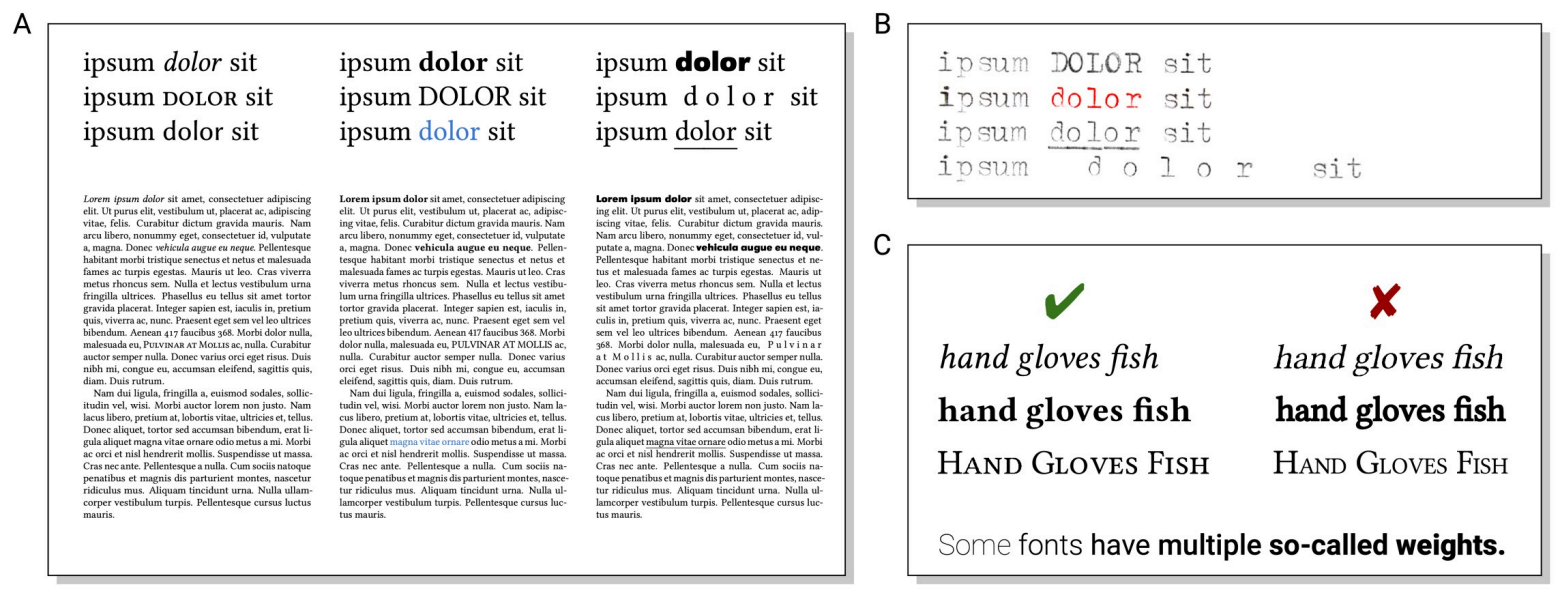
(A) Text in italics, small caps, or a matching alternative font does not change the type color of the page and emphasizes words within the context while reading. Bold text, uppercase letters, and different colors are more prominent and serve as “entry points” to the text. Using a contrasting alternative font, letter spacing, and underlining words forms an even stronger visual contrast, but is challenging to get looking good. (B) In the era of typewriters, authors were much more limited in using emphasis in their texts. (C) Italic, bold, and small caps should be used as properly designed font variants and not be faked by slanting (making the text look unnatural), making lines thicker (leading to, e.g., shrunken eyes and unbalanced spacing), or scaling capitals (making glyphs skinny). (Disclosure of image manipulation: text typed using a mechanical typewriter was digitized and edited for clarity, and color channels of the RGB image were manipulated to imitate red text from a 2-color ink ribbon).

Typographically, there are different variants of highlighting (Fig 5A), ranging from subtle to highly prominent. The prominence of emphasis can be characterized by the change of “color” [7] (or “type color” [31]), i.e., how dark the page appears at some location when viewed out of focus. Larger changes of type color are more prominent highlighting and easier to spot when just glancing at the page/poster/slide. Italic (no change in type color) is usually the formatting of choice for emphasis within context. In contrast, bold (notable change in type color) is useful, e.g., for headings or terms defined in a glossary. Small caps (no change in type color) are sometimes used to distinguish family names from given names or real-world from model entities. Underlining used to be one of the few possibilities of emphasis using a typewriter (see Fig 5B), but is neither particularly nice nor useful nowadays.

Emphasis in continuous texts should be used sparingly. If 80% of a text is emphasized, actually the remaining 20% of the text are most visible. In contrast, text not meant to be read as a whole may profit from extensively combining different ways of highlighting, e.g., markup in bibliographies or syntax highlighting in source code listings.

Pitfalls of highlighting are shown in Fig 5C. Italic, bold, and small caps of a font should only be used if available as properly designed variants. Automatically created variants (slanting glyphs, using thicker lines, or shrinking uppercase letters) are of lower quality (“Frankensteinian manipulation” [32]) and best avoided.

To achieve consistent visual emphasis throughout a document, suitable styles or macros should be defined and used. Naming these by purpose rather than appearance makes it easy to consistently change formatting when editing and revising a document (cf. Rule 9).

## Rule 5: Pages—Visually distribute your story

Unlike information on web pages, printed material and presentation slides are arranged on separate pages of fixed size. Contents thus need to be distributed with page breaks at useful locations (unless, of course, only a single page or a poster is needed). Besides text, also non-text material (figures, tables, and footnotes) needs to be positioned on pages.

For good readability, lines should not be longer than 75 to 80 characters [7] or require additional line spacing; otherwise, the readers’ eyes cannot easily jump from 1 line to the next. Reducing the margin width is thus not a good way to squeeze more content into a given number of pages. Also, margins are needed for the readers simply to hold the document without fingers covering part of the content and to take notes. Only little text such as page numbers should be placed in the margin (top outside, bottom outside, or bottom center). Two-column layout allows more readable text per page, but makes placing wide elements like figures or tables more complex. One-sided layout with page numbers at the bottom center is more robust if readers will likely print the document themselves and might not use duplexing or might print 2-on-1 (swapping left and right pages).

**Fig 6:**
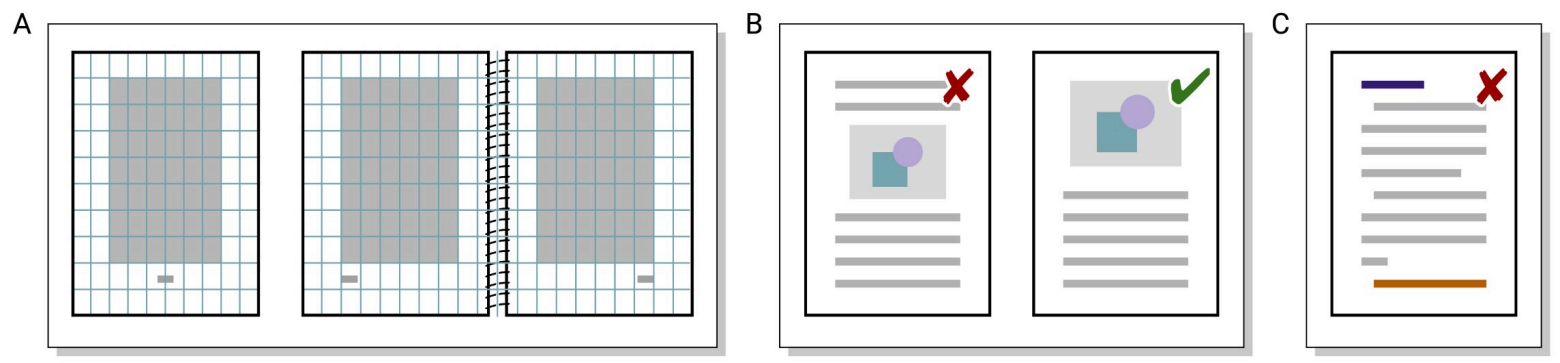
(A) One way to construct page margins for 1-sided and 2-sided layouts, also considering the type of binding used (here: spiral binding with narrower inner margin—glue or sewn binding requires enlarging the inner margin). (B) Figures (or tables) are commonly placed at the top of a page (or on figure-only pages), but not in between the text. (C) Orphans and widows, single lines of text separated from the rest of their paragraph, should be avoided.

If not defined by a template, one easy way [33] to define page margins (cf. Fig 6A) is to first determine how wide the text block needs to be to fit about 70 characters on average. The page is then divided into an *n*×*n* grid such that using 2 stripes of cells each as the left and right margins leaves the desired text width. One and 2 horizontal stripes are then used as the top and bottom margins, respectively. For 2-sided layout, each page should have only 1 stripe as the inner margin. In both cases, additional space for binding may need to be considered. For 12-point Cambria text on an A4-sized page, this construction results in *n* = 12 and margins of 35 mm, 25 mm, and 50 mm on the left/right, top, and bottom, respectively. In contrast, using a default setting of 1-inch margins for a letter-sized page containing 10-point Times New Roman text results in about 115 characters per line, too much for convenient reading.

Besides the running text, documents contain “floating” objects like figures or tables. These also need to be placed on the pages, typically at the top or bottom or on figure-only pages, ideally close to where they are referenced; see Fig 6B. Placing figures inside the running text would interrupt reading and may cause distracting page breaks. Instead, presenting figures in a separate “thread” outside the continuous text also permits readers to just browse through the figures and quickly find what interests them.

Footnotes, as the name suggests, are placed at the bottom of the page where they are referenced (which, clearly, should be done automatically). Footnotes are useful for relevant information complementing the main text without interrupting its flow (e.g., translations), see below for an example. Sometimes (e.g., to make presentation slides self-contained), footnotes are also used for literature references.

When optimizing page breaks, no single lines should be separated from the rest of a paragraph; see Fig 6C. Such single lines at the bottom and top of a page are denoted as widows (“have no future”) and orphans (“have no past”), respectively [33]. (The previous sentence is an example where a footnote would make sense: This vivid terminology is also used in other languages, e.g., the German terms for widow and orphan are “Hurenkind” [politically correct translation: offspring of a person working in the world’s oldest profession] and “Schusterjunge” [shoemaker’s apprentice].)

Text processing/typesetting software can places figures and partially prevent orphans and widows automatically, but this may require additional fine-tuning. Tricks for optimizing page breaks include rephrasing the text to make a paragraph on the affected page 1 line longer or shorter; enlarging the page vertically or breaking the page a line earlier; and moving, enlarging, or shrinking figures.

## Rule 6: Lists—Present some content in structured form

**Fig 7:**
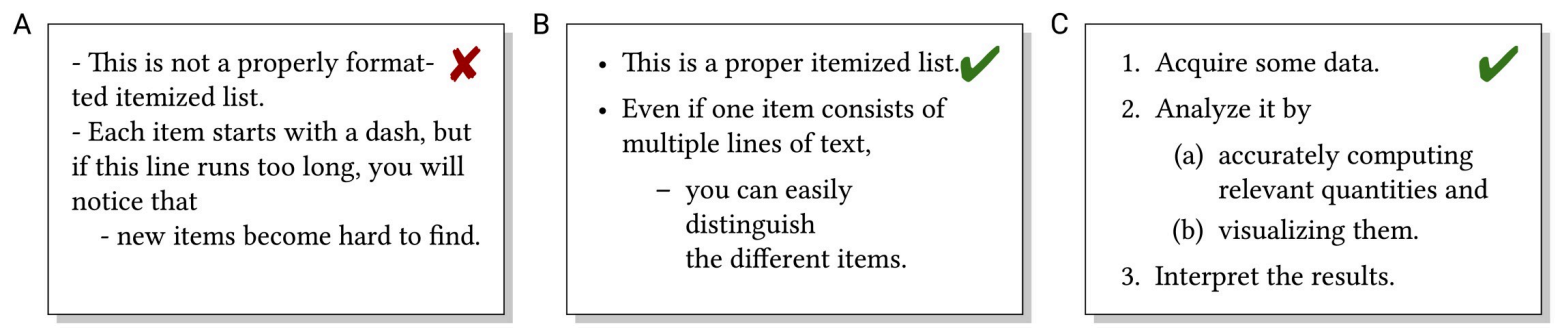
(A) Hard-to-recognize list, (B) properly formatted unsorted list, and (C) properly formatted numbered list.

Not all textual information is best presented as complete sentences in continuous text. In particular, as few text as possible should be used on slides [22,23] and posters [24,25]. Also in longer written texts, some information is best presented in (sub-)structured lists, either unsorted (itemized/bulleted) or sorted (numbered) lists; see Fig 7. The readability of lists may profit from manually optimizing line breaks, in particular on slides and posters.

Numbered lists are most useful for step-by-step instructions or if some of the entries are referenced from elsewhere (“item 5c” is more convenient than “the third sub-item of the fifth item”). Other common types of structured lists are glossaries (alphabetically sorted definition of terms where readers should quickly find the term they are looking for) and bibliographies (which additionally require cross-referencing from the main text). Itemized and numbered lists should be formatted consistently, i.e., they should be entered as the appropriate type of list and not by manually entering bullets/numbers and indentation; see Fig 7.

Particularly in bibliographies, it makes sense to conceptually distinguish content and layout. Here, the same information (author, title, journal, volume, year, etc.) should be printed in 1 consistent style (format, referencing from the text, and sorting). Using suitable reference management integrated with the text processing avoids manually formatting bibliographies.

## Rule 7: Figures, plots, and tables—Do not neglect the text outside the continuous text

Typography is relevant not only for the continuous text, but also for text in figures, plots, and tables. Figures convey content in easy-to-grasp graphical form, and plots present data in visual form, whereas tables provide precise numbers. Creating high-quality and well-readable figures [28,34–39] can be challenging, but is worth spending effort; well-designed figures with self-contained captions telling the main story are a useful way of reaching hurried readers just browsing through your work [15] or starting reading by looking at figures [40]. In particular, a good graphical abstract [41] or concept figure [42] can attract readers (even though the impact on citations is unclear [43]).

**Fig 8:**
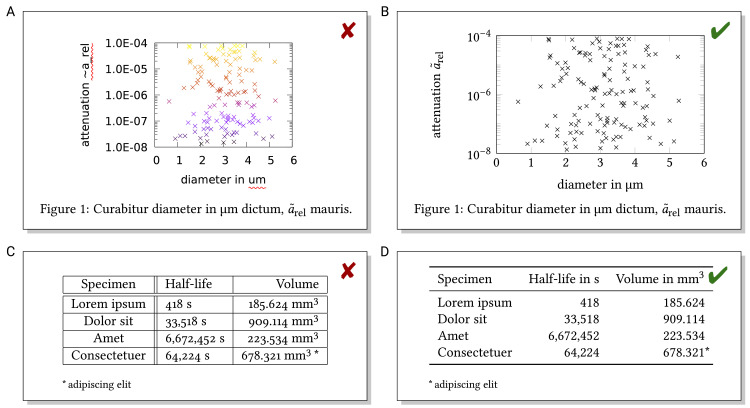
(A) Poor-quality plot: numbers are hard to read, symbols are not displayed properly, and color provides no additional information (except that automatic spell checking marked part of the axis labels as wrong). (B) Same data, better plot quality. (C) Poor-quality table: excessive lines and hard-to-read numbers, even in the right-aligned column due to the footnote symbol and numerals of different width. (D) Same information, better table layout.

Figures may contain different amounts of text that should be consistent with the main text not only in terminology, but also in terms of fonts and symbols. Figures are often created in separate software, so consistency may be challenging. However, the limited capability of software is not a convincing excuse for low-quality figures (cf. Fig 8A versus Fig 8B), and malicious readers could interpret it as limited capability of the author.

Table formatting includes proper column alignment. While text should be left-aligned in columns, numbers in columns can only be compared conveniently if printed right-aligned and written in numerals that are all of the same width (table figures). Tables should not include too many prominent lines to prevent the impression of a “prison cell” (cf. Fig 8C versus Fig 8D). Instead, tables can be structured optically by moderate spacing, light shading of every other row, or light lines. Whitespace is useful for structuring contents [44,45], elsewhere as well, e.g., in figures and lists (cf. Rule 6).

## Rule 8: Mathematical and chemical formulas—Do not let doubt enter the equation

Numbers should not only be correct, but should also be formatted appropriately. Numbers with more than 4 digits are grouped using commas between each group of 3 digits: 31,556,952. For decimal numbers, a period (“point”) is used as the decimal separator: 3.14. Following [10], ordinal numbers should be written as 1st, 2nd, 3rd, 4th, … without superscript letters. When reporting computer-generated results, notation like 5·10^−9^ is easier to read than pasting 5e−9 verbatim (and shows that you know what the “e” stands for).

**Fig 9:**
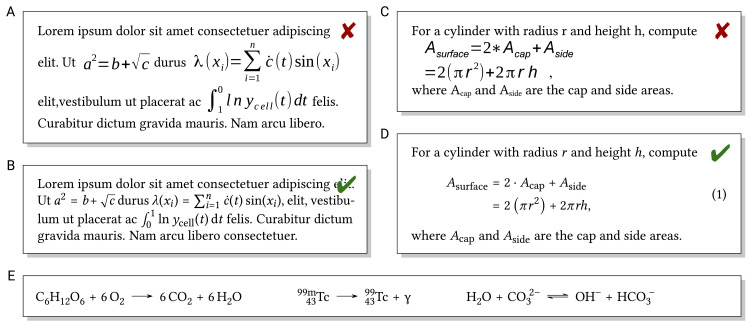
(A versus B) Formulas in the text should use a font matching the text and match the baseline of the text. Mathematical variables should be typeset in italic, unlike text parts of formulas or certain functions. (C versus D) Similarly, displayed formulas should match the surrounding text and are easier to understand if properly aligned. (E) Chemical formulas include subscript and superscript indices around symbols for chemical elements.

Formulas provide precise information in a very condensed form. They are difficult to get right in the first place, and incorrect typesetting can alter the meaning: consider, e.g., 2^3^ = 8 versus 23, or the isotope ^14^C versus 14 carbon atoms. Formulas are a particular example where correct typesetting is indispensable to show you understood what you have written. Shorter and simpler formulas can be included “inline” in the text (Fig 9A and 9B). In this case, font, font size, and the base line should match the surrounding text. More complex formulas, those to be cross-referenced by number or formulas too high to fit in the text without modified line spacing, are better written as displayed formulas (Fig 9C and 9D). From a grammar and punctuation perspective, also displayed formulas should be considered part of the sentences in the containing text. Depending on the text processing software used, formulas can be entered via math syntax or equation editors or (e.g., in case of complex chemical formulas) may need to be created in external software and imported as images.

In formulas, mathematical variables are commonly typeset in italic. However, mathematical functions like sin (sine), text (including, e.g., abbreviations in indices), units, chemical elements, and certain constants should not appear in italic (Fig 9B, 9D, and 9E). To make longer formulas easier to read, proper alignment and grouped brackets of matching size are helpful (Fig 9D).

## Rule 9: Use templates and styles for automatic and consistent formatting

When writing texts, unfinished layout may distract from content and structure. However, these topics should be addressed and concentrated on first, see Rule 10. Using software like Microsoft Word, LibreOffice Writer, or Google Docs that uses the “what you see is what you get” principle, layout needs to be ignored actively, unless a structure view, disabled page preview, or similar is used. Writing text in markup languages (e.g., Markdown or LaTeX using “what you see is what you mean”) makes the separation of content/structure and format/layout easier, but requires more technological affinity.

**Fig 10:**
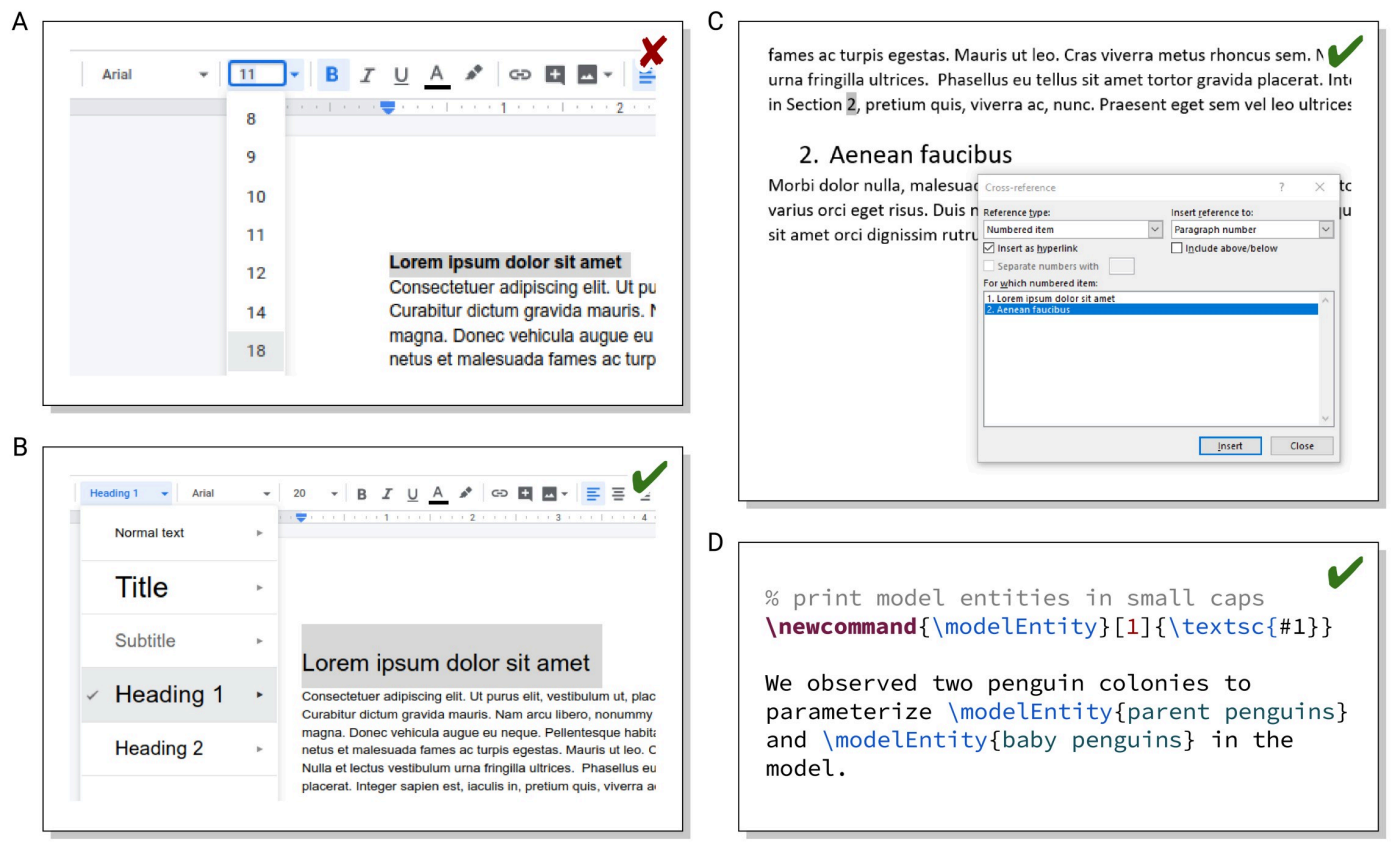
(A versus B) Marking headings as the appropriate level of headings (instead of manually formatting it bold and larger) ensures consistent layout (Google Docs example). (C) Using automatically numbered headings and proper cross-references (rather than manually entering it) allows keeping them up to date automatically (Microsoft Word example). (D) Using a macro name indicating its purpose (rather than having to remember the formatting for a specific purpose) makes writing easier (LaTeX example).

Structure in texts should be defined by styles/macros declaring, e.g., a section heading as a “level 1 heading” rather than manually numbering it, formatting it to a specific font size in bold with additional line spacing, making sure it is not followed by a page break, etc.; see Fig 10. Properly structuring in this way also permits automatically creating a table of contents, cross-referencing to section numbers without keeping them up to date manually, automatically using the same style, or conveniently switching the style if the template is changed. Similarly, figures and tables with captions should be included as such objects so that they can be positioned automatically at the top/bottom or on separate pages, again with the side effect of automatic numbering and cross-referencing.

## Rule 10: Iterative writing and typesetting—Do first things first and last things last

Formatting manuscripts is an iterative process, just like writing the contents [12,16]. When drafting contents at an early stage of the writing process, it only makes sense to pay attention to typographic issues that will likely be missed or cause problems/increased efforts later. This includes proper structuring, cross-referencing, and using template styles/macros. When editing the text later on, effort should be invested in those parts to be kept in the final manuscript. Issues relevant at this stage include, e.g., the contents of proper formulas, tables, and figures. Only when the contents have been finalized, it makes sense to polish the layout by optimizing line or page breaks and figure placement. Prematurely polishing either language or layout of parts of text that are deleted later is wasted effort.

Solitary and collaborative [18,20] writing may use a different format/platform than the one used for formatting and finalizing the submission, e.g., one may collaborate via a Google Doc or via Markdown files in a Git repository followed by finalizing the layout in LibreOffice Writer or LaTeX. Moreover, input from one or different authors needs to be unified also on a technical level, regardless of the technical platform used. Enough time should be planned for the work needed to turn finalized content into a formatted document ready for submission.
